# Use, Perspectives, and Attitudes Regarding Diabetes Management Mobile Apps Among Diabetes Patients and Diabetologists in China: National Web-Based Survey

**DOI:** 10.2196/12658

**Published:** 2019-02-08

**Authors:** Yiyu Zhang, Xia Li, Shuoming Luo, Chaoyuan Liu, Yuting Xie, Jia Guo, Fang Liu, Zhiguang Zhou

**Affiliations:** 1 Department of Metabolism and Endocrinology The Second Xiangya Hospital Central South University Changsha China; 2 Key Laboratory of Diabetes Immunology, Ministry of Education Changsha China; 3 National Clinical Research Center for Metabolic Diseases Changsha China; 4 Department of Oncology The Second Xiangya Hospital Central South University Changsha China; 5 Xiangya School of Nursing, Central South University Changsha China

**Keywords:** diabetes mellitus, mobile applications, surveys and questionnaires

## Abstract

**Background:**

The diabetes disease burden in China is heavy, and mobile apps have a great potential for diabetes management. However, there is a lack of investigation of diabetes app use among Chinese diabetes patients and diabetologists. The perspectives and attitudes of diabetes patients and diabetologists regarding diabetes apps are also unclear.

**Objective:**

Our objectives were to investigate diabetes patients’ and diabetologists’ use, attitudes, and perspectives, as well as patients’ needs, with respect to diabetes apps to provide information regarding the optimal design of diabetes apps and the best strategies to promote their use.

**Methods:**

Diabetes patients and diabetologists across China were surveyed on the WeChat (Tencent Corp) network using Sojump (Changsha ran Xing InfoTech Ltd) from January 23, 2018, to July 30, 2018. In total, 2 survey links were initially sent to doctors from 46 Latent Autoimmune Diabetes of Adults Study collaborative hospitals in China in 25 major cities and were spread on their WeChat contacts network. We also published the patient survey link on 3 WeChat public accounts and requested diabetes patients to fill out questionnaires. A multivariate regression analysis was used to identify associations of demographic and basic disease information with app usage among adult patients.

**Results:**

Overall, 1276 individuals from 30 provincial regions responded to the patient survey; among them, the overall app awareness rate was 29.94% (382/1276) and usage was 15.44% (197/1276). The usage was higher among patients with type 1 diabetes (T1DM) than among patients with type 2 diabetes (T2DM; 108/473, 22.8% vs 79/733, 10.8%; *P*<.001). The multivariate regression analysis showed that diabetes type, age, education, family income, and location were associated with app use in adult patients (*P*<.05). The need for and selection of diabetes apps differed slightly between patients with T1DM and patients with T2DM. The reasons why patients discontinued the use of an app included limited time (59/197, 29.9%), complicated operations (50/197, 25.4%), ineffectiveness for glycemic control (48/197, 24.4%), and cost (38/197, 19.3%).

Of the 608 responders to the diabetologist survey, 40.5% (246/608) recommended diabetes apps to patients and 25.2% (153/608) used diabetes apps to manage patients. The greatest obstacles to the diabetologists’ use of apps to manage diabetes patients include limited time (280/608, 46.1%), legal issues (129/608, 21.2%), patients’ distrust (108/608, 17.8%), and billing issues (66/608, 10.9%).

**Conclusions:**

The awareness and use of diabetes apps in Chinese people with diabetes and the proportion of diabetologists using diabetes apps to manage patients are low. Designing apps targeting different patient needs and conducting high-quality randomized controlled trials will improve the effectiveness of the apps, provide evidence for patients to choose suitable apps, and be conducive to the promotion of app use.

## Introduction

### Background

The prevalence of diabetes has been increasing worldwide. In 2017, it was estimated that there were 451 million people aged 18 to 99 years with diabetes. Furthermore, 5.0 million deaths were attributable to diabetes [1]. Among the Chinese adult population, the estimated prevalence of diabetes was 11.6%, representing an estimated 113.9 million adults in China with diabetes. However, only 39.7% of those treated had adequate glycemic control [2]. Poor glycemic control can cause various complications [3] and bring heavy economic burden to the world. In 2015, the global cost of diabetes was estimated to be US $1.31 trillion or 1.8% of the global gross domestic product (GDP) [4].

Diabetes self-management education and support (DSMES) is a critical element of care for patients with diabetes [5]. The AADE7 Self-Care Behaviors defined by the American Association of Diabetes Educators is a framework for patient-centered diabetes education and care [6]. The 7 self-care behaviors are eating healthy, being active, monitoring, taking medications, solving problems, healthy coping, and reducing risks, and these are essential for diabetes self-management. Due to an imbalance of medical resources in China [7], patients flock from rural areas to urban areas seeking medical resources. However, doctors in tertiary hospitals are overloaded with work [8,9]. Patients receive only a few minutes for outpatient consultation and receive little self-management knowledge in such a limited time. Furthermore, many outpatients do not have a record of their blood sugar; therefore, doctors cannot give accurate guidance for their treatment. Thus, a different type of health service might be needed to supplement traditional outpatient consultations.

Mobile apps can record, transmit, and receive feedback anytime and anywhere, facilitating remote monitoring and delivery of timely recommendations for health care. Mobile apps could increase the capacity for self-management, help sustain necessary lifestyle modifications, and improve communication between patients, family members, and health care professionals (HCPs). Furthermore, there were 1.32 billion mobile phone users in China in 2016 [10]. With the popularity of smart phones in China, mobile apps have great potential for managing chronic diseases, especially diabetes. A systematic investigation revealed that diabetes apps are the most common disease-specific apps in China’s mobile health (mHealth) market [11]. Many studies have demonstrated that diabetes apps can potentially help control glycemia [12-16]. Mobile apps are also recommended by the American Diabetes Association guidelines for DSMES [17]. A meta-analysis of 21 randomized controlled trials (RCTs) showed that diabetes apps were associated with a mean reduction of 0.57% in glycosylated hemoglobin (HbA_1c_) among patients with type 2 diabetes mellitus (T2DM) and 0.49% among patients with type 1 diabetes mellitus (T1DM). However, the results had significant heterogeneity [18]. Several studies also suggested that diabetes app use can increase blood glucose monitoring frequency [13,19], reduce feelings of loneliness, help patients gain knowledge and skills to manage diabetes [20], improve hypoglycemic fears and behavioral scores [15], and strengthen the perception of self-care by offering better information and health education to patients [16].

Diabetes app usage varies among different countries [21-25], from 3% in Latin countries in 2015 [21] to 19.6% in New Zealand in 2016 [22]. China has the largest absolute disease burden of diabetes in the world and the greatest potential diabetes app market. However, there is a lack of large-scale investigations of the usage of diabetes apps in China. Patients’ perspectives and attitudes regarding diabetes apps are also not very clear. In addition, user requirements are very important for app design. A survey by Boyle et al revealed that the most favored feature of patients was a glucose diary, and an insulin calculator was the most desirable function for a future app [22]. A survey by Trawley et al showed carbohydrate counting was the most common purpose among adults with T1DM and glucose monitoring was the most common purpose among adults with T2DM [23]. A recent meta-analysis of diabetes apps revealed that the reduction in HbA_1c_ is explained by the frequency of HCP feedback [18]. However, few surveys have been conducted to investigate the feature of patient-doctor communication. Our previous study found that both patients and diabetologists believed that doctor-patient communication and diabetes diaries were the most important functions of a diabetes app [26]. However, our previous study focused only on patients with T1DM and the samples were relatively small. Diabetologists’ use, attitudes, and perspectives concerning diabetes management apps in China are poorly understood.

### Objectives

The objective of our study was to investigate the use, perspectives, attitudes, and associated factors of diabetes patients and diabetologists regarding diabetes management apps, as well as patient needs for these apps, to provide information for the design of diabetes apps and how to best promote their use.

## Methods

### Questionnaire Design

The questionnaire design was described in a previous study [26]. An expert panel consisting of 5 diabetologists (YZ, SL, YX, XL, and ZZ) and a diabetes education nurse (FL) searched apps from the iOS and Android platforms and designed the questionnaires according to the functions of current diabetes apps [25,27-31], diabetes guidelines [32], and the problems they encountered during clinical practice. The questions were presented in a choice format. If responders did not agree with the listed options, they could select the option *others* and write their answers in the remarks column. The diabetologist questions covered their basic information and use, attitudes, and perspectives regarding diabetes management apps. The patient questions covered their use, perspectives, and attitudes regarding and needs for diabetes apps, demographic information, and basic disease information.

To establish the validity of the content, the survey items were rated based on their relevance and clarity using a 4-point ordinal scale from 1 (irrelevant) to 4 (highly relevant) by 15 experts: 12 diabetologists and 3 diabetes education nurses with at least 5 years of experience treating patients with diabetes. The content validity indexes of the patient and diabetologist questionnaires were 0.91 and 0.93, respectively. Before administering the questionnaires, we performed pilot tests with 20 patients with diabetes and 10 diabetologists from Second Xiangya Hospital. As 3 patients were unwilling to disclose their exact income and 1 doctor was reluctant to reveal his age, we revised our questionnaires such that patients were not required to answer the question regarding income and the doctors’ ages were grouped into ranges. Cronbach alpha values for the patient and diabetologist questionnaires were .98 and .79, respectively.

### Introduction of the WeChat Survey Platform

WeChat provides many services, including messaging, free phone calls, browsing and posting for information sharing on moments, and mobile payments [33]. It is installed in over 90% of mobile phones and is integrated into most people’s daily lives [34]. As the most widely and frequently used social communication tool in China, WeChat has a powerful contact network. As of 2016, over 44% of WeChat users had more than 200 contacts on the social networking service. Approximately 90% of WeChat users had more than 50 contacts [35]. This network makes it possible to administer questionnaires via WeChat.

### Samples and Survey Methods

The participants were diabetes patients and diabetologists across China. Doctors at other departments who did not treat diabetes patients were excluded from our investigation.

Diabetes patients and diabetologists were surveyed through snowball sampling via the WeChat contacts network and convenience sampling through WeChat public accounts using the Web-based survey tool, Sojump, [36] from January 23, 2018, to July 30, 2018. The patient and diabetologist survey links were initially sent to doctors from 46 latent autoimmune diabetes of adults (LADA) Study China collaborative hospitals in 25 representative major cities in China [37]. We asked these doctors to spread the survey links on their WeChat contacts network. In addition to using snowball sampling through the WeChat contacts network, we published the patient survey link on 3 WeChat public accounts concerning diabetes, which have 50,000 subscribed followers, and asked diabetes patients to complete the questionnaires. The parents of juvenile patients answered the questions for their children. We introduced the background of our survey, and the questionnaires were completed voluntarily without any compensation. A part of the survey results concerning patients with T1DM has been reported in a previous study [26].

### Ethical Approval

The study was approved by the ethics committee of the Second Xiangya Hospital, Central South University (ID: 2017-S107).

### Statistics

The data were analyzed using SPSS version 23.0 (IBM Corp). Q-Q plots were used to check the normality of all the continuous variables, which are expressed as the means (SDs) or medians (interquartile ranges [IQRs]) where appropriate. Categorical variables are expressed as percentages (numbers, n). Differences among groups were assessed using Chi-square tests. The generalized logistic model was used to obtain odds ratios (OR) and their 95% CIs in a simultaneous manner. First, we performed a univariable analysis to obtain unadjusted ORs of potential correlates of app use with demographic factors and disease characteristics in adult patients (aged ≥18 years). We then entered all the significant factors in the multivariate analysis to obtain the multivariable adjusted ORs. Questionnaires with missing values were excluded from the multivariate analysis. Statistical significance was indicated with *P*<.05.

## Results

### Patient Survey

#### Sample Characteristics

**Figure 1:**
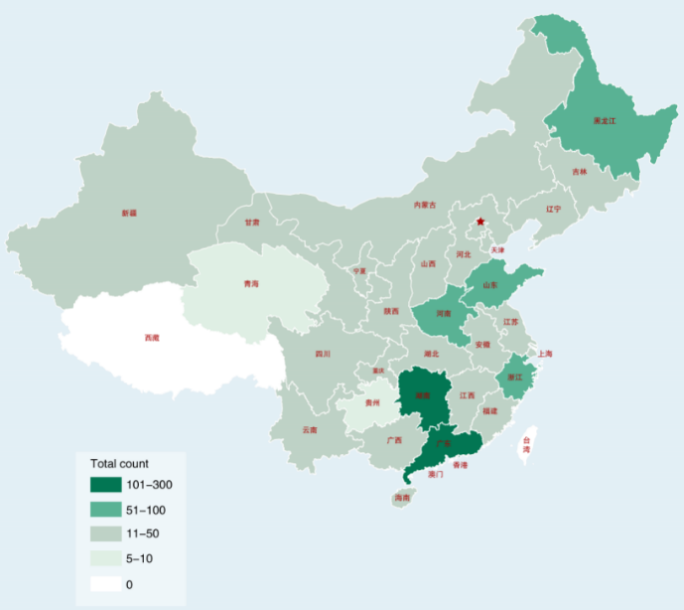
Distribution of the diabetic patient sample in China by province.

A total of 1276 patients with diabetes (414 from North China and 862 from South China) distributed among 30 provinces in China (Figure 1) responded to the patient survey. The responder characteristics are shown in Table 1. Of the responders, 50.31% (642/1276) were male, with a mean age of 41.3 years (SD 18.5). The mean disease duration was 6.8 years (SD 6.9). Overall, 37.07% (473/1276) were patients with T1DM and, of these, 178 were juveniles; 57.45% (733/1276) were patients with T2DM; 2.12% (27/1276) of the patients had gestational diabetes; and 3.45% (43/1276) did not know their diabetes type.

**Table 1:** Characteristics of patients with diabetes.

Characteristics		T1DM^a^ (n=473)		T2DM^b^ (n=733)	Total^c^ (N=1276)
		Adults (n=295)	Juveniles (n=178)		
**Gender, n (%)**					
	Male	115 (39.0)	81 (45.5)	426 (58.1)	642 (50.31)
Age (years), mean (SD)		33.5 (11.9)	10.3 (4.2)	52.2 (12.0)	41.3 (18.5)
Disease duration (years), mean (SD)		7.9 (8.0)	3.0 (3.2)	7.7(6.8)	6.8(6.9)
**Residence, n (%)**					
	Urban	206 (69.8)	110 (61.8)	572 (78.0)	933 (73.12)
	Rural	89 (30.2)	68 (38.2)	161 (22.0)	343 (26.88)
**Education, n (%)**					
	Junior middle school or below	55 (18.6)	—^d^	193 (26.3)	422 (33.07)
	High school	93 (31.5)	—	244 (33.3)	373 (29.23)
	University or above	147 (49.8)	—	296 (40.4)	481 (37.70)
**Treatment, n (%)**					
	Oral medicine	14 (4.7)	1 (0.6)	379 (51.7)	416 (32.60)
	Insulin injection	196 (66.4)	128 (71.9)	281 (38.3)	636 (49.84)
	Insulin pump	83 (28.1)	49 (27.5)	9 (1.2)	144 (11.29)
	Untreated	2 (0.7)	0	64 (8.7)	80 (6.27)
**Occupation, n (%)**					
	Student	44 (14.9)	—	3 (0.4)	206 (16.14)
	Institutional staff	45 (15.3)	—	115 (15.7)	171 (13.40)
	Employee of state-owned enterprise	19 (6.4)	—	83 (11.3)	109 (8.54)
	Employee of foreign or private company	43 (14.6)	—	69 (9.4)	121 (9.48)
	Private enterprise owner or self-employed	26 (8.8)	—	77 (10.5)	113 (8.86)
	Retired	19 (6.4)	—	206 (28.1)	231 (18.10)
	Farmer	19 (6.4)	—	75 (10.2)	103 (8.07)
	Unemployed	47 (15.9)	—	70 (9.5)	131 (10.27)
	Others	33 (11.2)	—	35 (4.8)	9 (7.05)

^a^T1DM: type 1 diabetes mellitus.

^b^T2DM: type 2 diabetes mellitus.

^c^Total including patients with T1DM, patients with T2DM, 27 patients with gestational diabetes, and 43 patients with an unknown type of diabetes.

^d^Indicates that there is no value.

#### Diabetes App Use and Associated Factors Among Diabetes Patients

The overall diabetes app awareness rate was 29.94% (382/1276), the usage rate was 15.44% (197/1276), and 43.7% (86/197) of the patients who use the apps used them every day. The app usage of patients with T1DM was higher than that of patients with T2DM (108/473, 22.8% vs 79/733, 10.8%; *P*<.001). The utilization rate of adult patients with T1DM was higher than that of juvenile patients with T1DM (80/295, 27.1% vs 28/178, 15.7%; *P*=.004). A comparison of patients located in the 10 provinces of top GDP per capita [38] with the other 20 provinces showed that the former had higher diabetes app usage than the latter (79/401, 19.7% vs 118/875, 13.5%; *P*=.004).

The app usage of juvenile patients with T1DM treated with an insulin pump was higher than that of those treated with a subcutaneous injection (13/49, 26.5% vs 15/128, 11.7%; *P*=.009). The comparison of the 3 groups of juvenile patients with different parental educational levels (junior middle school and below, senior high school, and university and above) revealed that a higher education level of parents was associated with a higher app usage (4/64, 6.3% vs 6/41, 14.6% vs 17/65, 26.2%; *P*=.008). App usage in children was higher than that in adolescents, but the difference was not statistically significant (21/106, 19.8% vs 7/72, 9.7%; *P*=.07).

A univariate regression analysis showed that app use by adult patients was significantly associated with age, annual family income, occupation, education, locus, type of diabetes, and treatment (*P*<.05). Gender, disease duration, and rural or urban residence had no correlation with app use. Variables with statistical significance were included in the multivariate regression analysis. Diabetes type, age, education, annual family income, and location remained statistically significant (*P*<.05). Compared with low-income patients, patients with high income had higher app usage (OR=1.73, 95% CI 1.07-2.81; *P*=.03). The use of apps decreased with patient age. Notably, the higher the education level of the patients, the higher was the usage of apps (see Table 2).

#### Patients’ Need for and Selection of Diabetes Apps

**Figure 2:**
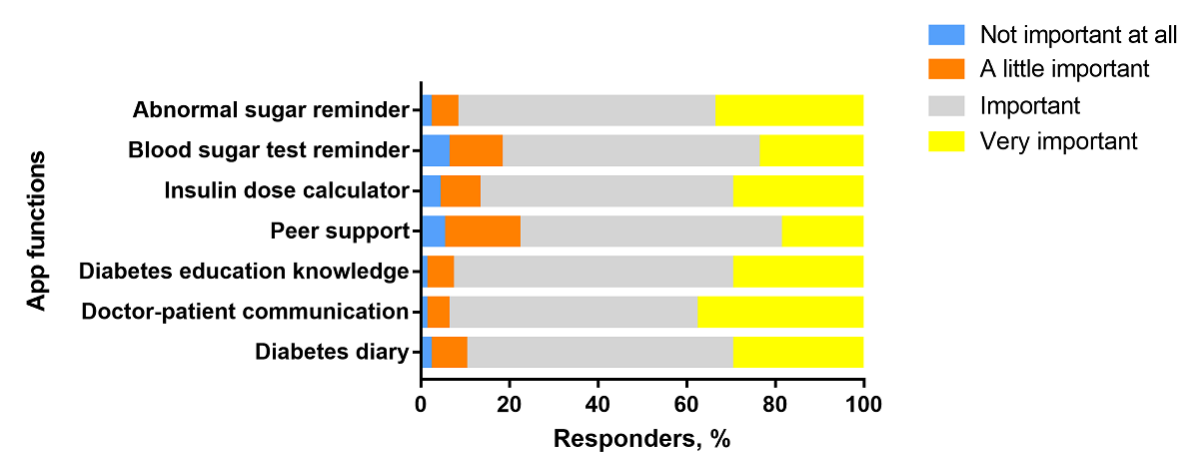
Importance of different app functions reported by patients with diabetes (N=1276).

Patients with both T1DM and T2DM believed that the most important functions of a diabetes app were diabetes diaries (blood sugar, diet, exercise, and medication records) and doctor-patient communication (Table 3). Patients with T2DM had a greater demand for doctor-patient communication (299/733, 40.8% vs 151/473, 31.9%; *P*=.002), and patients with T1DM had a greater demand for an insulin dose calculator (51/473, 10.8% vs 11/733, 1.5%; *P*<.001). Almost all patients believed that the listed functions were important or very important (Figure 2).

**Table 2:** Factors associated with app use by multivariate logistic regression analysis (N=1008).

Characteristics		App usage rate, n (%)	Adjusted odds ratio (95% CI)	*P* value
**Age (years)**				
	18-39^a^	97 (27.2)	—^b^	—
	40-59	47 (10.7)	0.42 (0.27-0.65)	<.001
	≥60	19 (9.0)	0.40 (0.22-0.72)	.002
**Annual family income^c^**				
	¥<50,000^a^	34 (10.9)	—	—
	¥50,000-100,000	50 (16.1)	1.38 (0.84-2.28)	.20
	¥>100,000	79 (20.5)	1.73 (1.07-2.81)	.03
**Diabetes type**				
	T2DM^a,d^	78 (11.2)	—	—
	T1DM^e^	76 (29.7)	2.0 (1.30-3.05)	.001
	Gestational diabetes	3 (11.1)	0.47 (0.13-1.67)	.25
	Unknown type	6 (16.2)	0.15 (0.45-2.93)	.77
**Location**				
	The other 20 provinces^a^	96 (14.1)	—	—
	10 provinces of the top GDP^f^ per capita	67 (20.5)	1.5 (1.04-2.17)	.03
**Education**				
	Junior middle school or below^a^	18 (7.6)	—	—
	High school	49 (15.0)	1.85 (1.02-3.37)	.04
	University or above	96 (21.7)	2.40 (1.34-4.25)	.003

^a^Reference group.

^b^Not applicable.

^c^90 samples with missing data on family income were excluded from the logistic regression analysis.

^d^T2DM: type 2 diabetes mellitus.

^e^T1DM: type 1 diabetes mellitus.

^f^GDP: gross domestic product.

**Table 3:** App functions considered to be most important by patients with both T1DM and T2DM.

Features	T1DM^a^ (N=473), n (%)	T2DM^b^ (N=733), n (%)	*P* value ^c^
Diabetes diaries	109 (23.0)	192 (26.2)	.22
Doctor-patient communication	151 (31.9)	299 (40.8)	.002
Diabetes education knowledge	54 (11.2)	86 (11.7)	.87
Peer support	13 (2.7)	10 (1.4)	.09
Insulin dose calculator	51 (10.8)	11 (1.5)	<.001
Abnormal blood sugar reminder	60 (12.7)	85 (11.6)	.57
Blood sugar test reminder	8 (1.7)	15 (2.0)	.66
Others	27 (5.7)	35 (4.8)	.47

^a^T1DM: type 1 diabetes mellitus.

^b^T2DM: type 2 diabetes mellitus.

^c^A Chi-square test was used to calculate *P* values.

Differences in app selection were found between patients with T1DM and patients with T2DM (see Table 4). The most popular app among patients with T1DM was Diabetes Circle (30/108, 27.8%), which is targeted for patients with T1DM, followed by Diabetes Nurse (28/108, 25.9%) to which blood sugar tested by a Dnurse glucometer (Sinocare Inc) can be directly transmitted. The most popular app among patients with T2DM was Diabetes Nurse (34/79, 43.0%).

Only 19.3% (38/197) of the apps were recommended by HCPs. Most patients selected diabetes apps as recommended by other patients (55/197, 27.9%) or selected randomly because they did not know which one was best (54/197, 27.4%). The rest were recommended by the media (30/197, 15.2%) or other channels (20/197, 10.2%).

**Table 4:** Differences in the selection of diabetes apps between patients with type 1 diabetes mellitus (T1DM) and patients with type 2 diabetes mellitus (T2DM).

App name	T1DM (N=108), n (%)	T2DM (N=79), n (%)	*P* value^a^
Welltang (Shanghai Geping Information Technology Co, Ltd)	24 (22.2)	5 (6.3)	.003
Diabetes Circle (Aibaowei Biotechnology Co, Ltd)	30 (27.8)	3 (3.8)	<.001
Control Diabetes (Fuzhou Kangwei Network Technology Co, Ltd)	1 (0.9)	5 (6.3)	.08
Diabetes Doctor (Shanghai Huima Medical Technology Co, Ltd)	6 (5.6)	14 (17.7)	.008
Diabetes Nurse (Beijing Dnurse Technology Co,Ltd)	28 (25.9)	34 (43.0)	.01
Others	19 (17.6)	18 (22.8)	.38

^a^A Chi-square test was used to calculate the *P* values.

#### Patients’ Perspectives on Diabetes Apps

Among the patients who had used diabetes apps, the reasons for discontinuation of use included limited time (59/197, 29.9%), complicated operations (50/197, 25.4%), ineffectiveness for glycemic control (48/197, 24.4%), cost (38/197, 19.3%), and others (48/197, 24.4%). Most patients thought that the diabetes app was very helpful (89/197, 45.2%) or helpful (67/197, 34.0%) to them. Overall, 14.2% (28/197) of the patients thought it was a little helpful. Only 6.6% (13/197) of them thought it was unhelpful.

Of the patients, 58.4% (115/197) indicated that their apps had a function for consulting HCPs and, of these patients, 50.4% (58/115) had used the function to consult an HCP. The proportion of patients with T2DM who consulted HCPs was higher than that of patients with T1DM, but this difference was not statistically significant (26/46, 57% vs 28/61, 46%; *P*=.28). A total of 67% (39/58) of their consultations were free. Most patients thought that these consultations were helpful (33/58, 57%) or very helpful (4/58, 7%). The reasons cited by the patients as affecting the effectiveness of the consultation included short consultation time (25/58, 43%), delayed response (15/58, 26%), unqualified HCPs (10/58, 17%), and others (8/58, 14%).

#### Patients’ Attitudes Toward Diabetes Apps

Only 34.87% (445/1276) of patients believed that consulting HCPs via apps should cost money. However, 59.72% (762/1276) of these indicated that they would continue to use this function if the consultation effect was good. Of these, 8.30% (106/1276) said they would certainly continue to use and 31.97% (408/1276) said they would not continue to use the app if they had to pay. Almost all patients said they were in need (889/1276, 69.67%) or in great need (335/1276, 26.25%) of a good app to help with their glycemic control. Only 4.08% (52/1276) of them said they did not need a diabetes app.

### Diabetologist Survey

#### Diabetologists’ Recommendation and Use of Diabetes Management Apps and Associated Factors

In total, 608 diabetologists (223 from North China and 385 from South China) from 21 provinces in China responded to the diabetologist survey. Table 5 shows the characteristics of the surveyed diabetologists.

**Table 5:** Characteristics of the surveyed diabetologists (N=608).

Characteristics		Statistics
**Gender, n (%)**		
	Male	197 (32.4)
	Female	411 (67.6)
**Age (years), n (%)**		
	≤30	99 (16.3)
	30-39	274 (45.1)
	40-49	162 (26.6)
	50-59	67 (11.0)
	≥60	6 (1.0)
**Title, n (%)**		
	Resident	141 (23.2)
	Attending specialist	239 (39.3)
	Associate chief doctor	125 (20.6)
	Chief doctor	103 (16.9)
**Hospital level, n (%)**		
	Tertiary hospital	419 (68.9)
	Secondary hospital or lower	189 (31.0)

**Figure 3:**
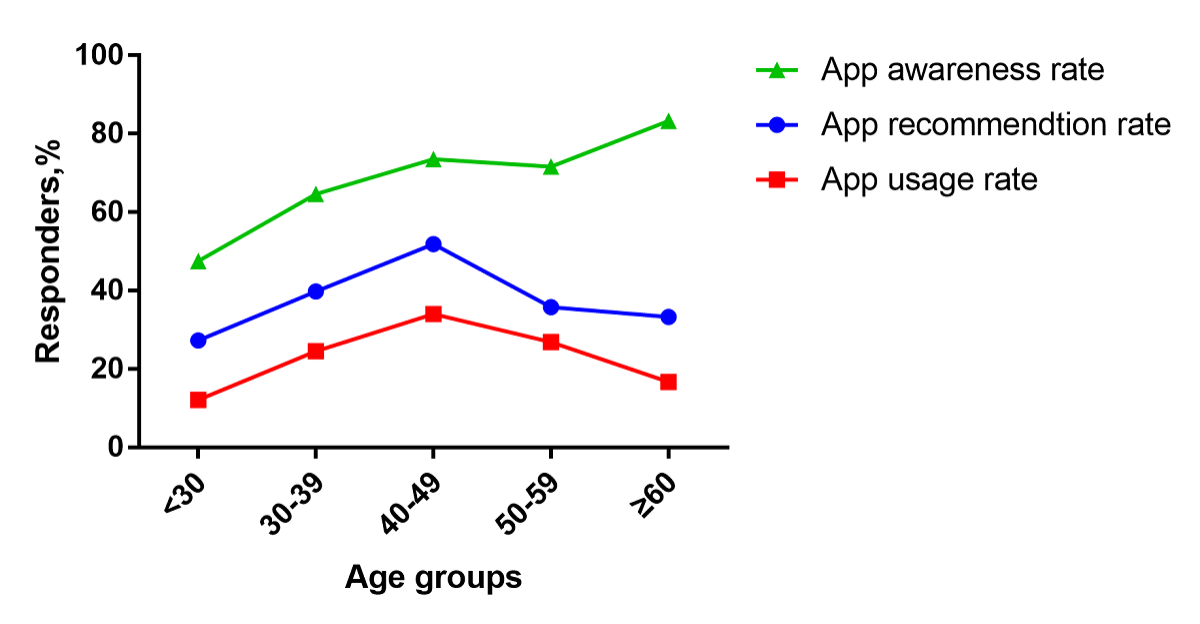
App awareness rate, recommendation rate, and usage among different age groups of diabetologists (N=608).

Of the diabetologists, 43.8% (266/608) had downloaded a diabetes management app, 40.5% (246/608) of them had recommended diabetes apps to their patients, and 25.2% (153/608) used diabetes apps to manage their patients. The app awareness rate was lowest in the doctor group younger than 30 years (47/99, 47%) and it gradually increased with age (*P*<.001). App recommendation rate and usage increased gradually with the age of doctors. The highest recommendation rate and usage were found in the 40 to 49 age group (84/162, 51.9% and 55/162, 34.0%, respectively), and then decreased again with age (*P*=.002 and *P*=.003, respectively; Figure 3). The app recommendation rate and usage among doctors in tertiary hospitals were higher than those in secondary or lower hospitals (190/419, 45.3% vs 56/189, 29.6%; *P*<.001 and 119/419, 28.4% vs 34/189, 18%; *P*<.001).

The app most recommended by diabetologists was Diabetes Doctor (56/246, 22.8%), which enables doctors to follow their patients, followed by Welltang (45/246, 18.3%), which is the only app tested via an RCT in China. The most important factors that influenced diabetologists’ recommendations of apps to their patients included not knowing of a suitable app (296/608, 48.7%), not knowing of the existence of diabetes apps (212/608, 34.9%), no time to recommend (182/608, 29.9%), no evidence demonstrating their effectiveness (90/608, 14.8%), no effect on blood sugar (47/608, 7.7%), and others (49/608, 8.1%).

The greatest obstacles to diabetologists’ use of apps to manage patients with diabetes include limited time (280/608, 46.1%), legal issues (129/608, 21.2%), patients’ distrust (108/608, 17.8%), uncertainty on how to bill patients (66/608, 10.9%), and others (25/608, 4.1%). The proportion of diabetologists in tertiary hospitals who thought the largest obstacle was limited time was higher than that in secondary or lower hospitals (212/419, 50.6% vs 68/189, 36.0%; *P*=.001). The proportion of doctors who believed that the greatest obstacle was patient distrust was higher in doctors from secondary or lower hospitals and junior doctors than in doctors from tertiary hospitals and among senior doctors (47/189, 24.9% vs 61/419, 14.6%; *P*=.002; and 38/141, 27.0% vs 70/467, 15.0%; *P*=.001, respectively).

In all, 94% (141/150) of the diabetologists use apps to manage patients free of charge. Most of them managed less than 50 patients (125/150, 83.3%). Most diabetologists who had managed patients with an app thought that the app had some effect (83/150, 55.3%) or minimal effect (58/150, 38.7%) on blood sugar, whereas 2.0% (3/150) thought it had no effect and 4% (6/150) thought it was very effective. Most diabetologists did not know whether it was legal to use apps to manage patients (311/608, 51.2%), and 33.6% (204/608) and 15.3% (93/608) thought that using an app for this purpose was legal and illegal, respectively.

#### Diabetologists’ Perspectives of Diabetes Apps

**Figure 4:**
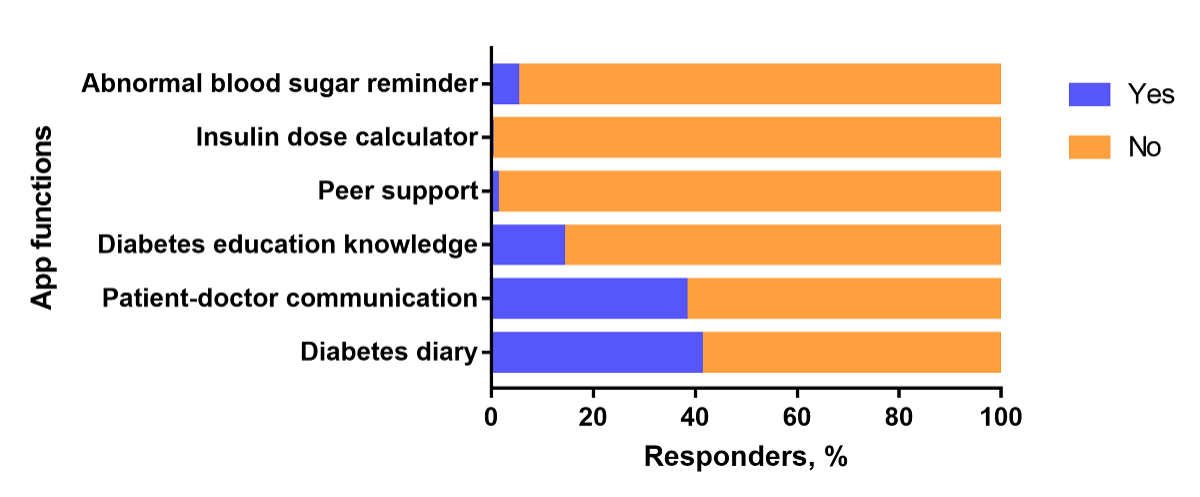
Diabetes app functions considered to be most important by diabetologists (N=608).

The diabetologists believed that the most important functions of a diabetes app were diabetes diaries (247/608, 40.6%) and doctor-patient communication (233/608, 38.4%; Figure 4). Of the diabetologists, 71.5% (435/608) believed that patients with T1DM and patients with T2DM needed different apps.

Diabetologists believed that the reasons for the poor effect of a diabetes app on blood sugar included the following: patients could not adhere to the use of apps (431/608, 70.9%); HCPs did not participate in apps or gave too little guidance for patients (383/608, 63%), diabetes education knowledge on an app was not systematic (280/608, 46.1%), apps lacked comprehensive functions (225/608, 37.0%), and others (16/608, 2.6%).

#### Diabetologists’ Attitudes Toward Diabetes Apps

Most diabetologists said they might (380/608, 62.5%) or would certainly (219/608, 36%) use a diabetes app to manage patients in the future. Only 1.5% (9/608) indicated that they would not manage diabetes patients using an app. Most said that they might (349/608, 57.4%) or would definitely (253/608, 41.6%) recommend diabetes apps to their patients, whereas only 1.0% (6/608) said that they would not recommend one. Most diabetologists believed that diabetes apps showed good (325/608, 53.5%) or very good (127/608, 20.9%) potential.

## Discussion

### Principal Findings

#### Diabetes App Use among Diabetes Patients and Its Associated Factors

App usage was 22.8% among patients with T1DM and 10.8% among patients with T2DM, which was comparable with surveys conducted in New Zealand [22] and Australia [23], and higher than the 7% found in a Scottish survey in 2016 [25] and the 3% found among Latinos in 2015 [21]. The app usage of patients with T2DM in China was lower than that of patients with T1DM and was associated with age, education, family income, and location. The possible reasons are that younger patients and well-educated patients are more likely to acquire and accept new technology, and those with higher household income are more likely to focus on their glycemic control and actively seek new ways to control blood sugar. China’s economic development is unbalanced, and medical resources are unevenly distributed and relatively concentrated in economically developed areas [7]. There are obvious regional differences in glycemic control in China [39]. The regional difference in app usage might be related to these differences in glycemic control. Furthermore, the app usage among patients with T2DM was lower than that among patients with T1DM, which is consistent with former studies [22,23]. This finding may be because the blood sugar of patients with T1DM is more difficult to control and their need for an app is greater.

We found that the app usage in children was higher than that in adolescents, possibly because children’s blood sugar is always managed by their parents, as their parents use diabetes apps to help with glycemic control. However, adolescents gradually withdraw support from their parents and take over the management tasks.

#### Suggestions for Promoting Diabetes App Use

App use among patients with diabetes in China is low, largely because of the low awareness of diabetes apps. Only 29.94% of the patients knew that diabetes apps existed, but only half of these patients who knew of diabetes apps would use one. Additionally, many doctors, particularly younger doctors, had no information on diabetes apps. Specifically, younger doctors had less awareness of diabetes apps than did senior doctors, which might be related to younger doctors having less awareness of the progress made in diabetes treatment. Therefore, public awareness of diabetes apps must be increased.

Our study found that only a small number of patient apps were recommended by doctors. Most apps were recommended by patients or were casually chosen. In total, 40.5% of the diabetologists in China recommended diabetes apps to their patients, which was lower than the 60.1% found in the New Zealand survey [22] and the 62% found in the US survey [40]. The most important factor that influenced diabetologists’ recommendation of apps to patients was that they had no idea of a suitable one among the numerous apps. Although there are thousands of diabetes apps, only a small number of them were tested for efficacy [31]. The quality of the studies was not high, and the effects of the apps on blood sugar were inconsistent [41]. Thus, it is difficult for HCPs to recommend a suitable app to their patients. Therefore, it is very important to carry out high-quality RCTs to test app efficacy [42].

#### Barriers to Doctor-Patient Communication and Suggestions for Improvement

Both diabetologists and patients believed that doctor-patient communication and diabetes diaries were the most important functions of a diabetes app, which was consistent with our previous report [26]. The app most recommended by diabetologists was one that doctors could use to follow-up with their patients. However, only 25.2% of the diabetologists managed diabetes patients with apps.

The main reasons given by diabetologists as affecting patient management with apps were limited time, issue of legality, and patients’ distrust. More doctors in tertiary hospitals than in primary hospitals thought that the largest obstacle to using an app to manage patients with diabetes was limited time, and more younger doctors and doctors from primary hospitals believed that patients’ distrust was the largest obstacle. Therefore, improving the specialty of young doctors and doctors from primary hospitals can effectively improve patients’ trust in these doctors, reduce the burden on senior doctors from tertiary hospitals, and effectively promote using diabetes apps to manage patients. Fortunately, the Chinese government is actively promoting standardized training for residents and a hierarchical medical system, which will effectively reduce the burden on doctors from tertiary hospitals, improve the specialties of young doctors and doctors from primary hospitals, and enhance patients’ trust in them. Owing to a lack of face-to-face physical examinations and complete medical histories, most doctors did not know whether using an app to manage patients was legal. China is vigorously promoting internet health care and improving relevant legislations [43]. Therefore, it may become possible for doctors to manage their patients with an app.

The approach used to bill patients was also a reason given by diabetologists that affected patient management. At present, most diabetologists use apps to manage patients free of charge, which affects the HCP’s enthusiasm. Medical insurance should be included, and an effective billing system should be established.

#### Suggestions for the Design of Diabetes Apps

There were a few differences in the needs of an app between patients with T1DM and patients with T2DM. More patients with T1DM believed that the insulin dose calculator was the most important function of diabetes apps. Patients with T1DM rely on insulin therapy, and insulin dosage must be adjusted according to diet and exercise. Thus, these patients have a greater need for this function. There were also differences in the choice of apps between the 2 groups. The most common choice of patients with T1DM was an app targeted for patients with T1DM (Diabetes Circle). The most common choice of patients with T2DM was Diabetes Nurse, to which blood sugar tested by a Dnurse glucometer can be directly transmitted. Additionally, most doctors believed that patients with T1DM and patients with T2DM need different apps. Therefore, apps should be designed according to different types of diabetes patients’ demands.

The data entry burden is the major reason why patients cannot persist in using an app [44]. Data transferred directly from a glucometer to an app will reduce the data entry burden, which is why most patients chose the Diabetes Nurse app. Diabetologists believed that the main reason why an app was ineffective was that patients could not persist in using it. The main reasons why patients did not want to continue to use an app were lack of time and complicated operations. Therefore, app design should enable blood sugar data from glucometers to be automatically transmitted to the app, which will greatly increase patient compliance.

Of the diabetologists, 46.1% believed that a lack of systematic and standardized diabetes education knowledge was a reason for the poor efficacy of apps. Most apps do not have educational information cited from accredited sources [45]. Previous studies indicated that a mobile app was preferable to receiving DSMES in a hospital [26,46]. Therefore, diabetes education knowledge compiled by a multidisciplinary team will effectively improve patients’ self-management ability and improve the effectiveness of an app.

#### Prospect of Diabetes Apps

Almost all patients indicated that they needed an effective app to manage blood sugar. Almost all diabetologists said they would or could use diabetes apps to manage patients and recommend diabetes apps to their patients. China has the largest number of patients with diabetes in the world [2], and our results suggest that diabetes apps have a very good future in China.

### Comparison With Prior Work

To our knowledge, no large-scale investigations have been conducted on diabetes app use among people with diabetes in China. An Australian survey suggested that disease duration was related to the use of app in diabetes patients [23,24]. Our study did not find such an association, probably because of different samples. The Australian survey only investigated patients with a disease duration of more than 1 year, and our study included patients of all disease durations. Furthermore, the disease durations were self-reported and might be inaccurate. Our study found that diabetes app users tended to be younger, have higher incomes, and be more educated, which was consistent with a health app survey in the United States [44].

Boyle et al investigated both diabetes patients and HCPs for their use and beliefs about diabetes apps. They also found that diabetes diaries were most useful for diabetes patients [22]. Our strength was that we recruited a large sample throughout China, and we also investigated the patient-doctor communication feature from the perspectives of both patients and doctors, which is considered to be the most important function by both patients and diabetologists [26].

Few studies have surveyed diabetes app use from the point of view of HCPs. Karduck and Chapman-Novakofski investigated clinicians’ perspectives on mHealth apps and found that most clinicians (62%) recommended mobile phone apps to their patients to track their diet and physical activity [40]. Our study found a 40.5% recommendation rate. However, most of their samples were nutritionists and diabetes educators. We recruited only diabetologists because diabetes patients in China are mostly treated by these doctors.

In addition to investigating patients’ app use, we also investigated diabetologists’ use, perspectives, and attitudes regarding diabetes apps, as well as patients’ needs, perspectives, and attitudes regarding diabetes apps. Therefore, we can provide more effective information for app design and promotion of app usage and better understand the diabetes app market in China.

### Limitations

A strength of our study was that the patient and diabetologist survey links were initially sent to doctors from 46 LADA China Study collaborative hospitals in 25 representative major cities and spread on their WeChat contact networks. In addition to this snowball sampling method, patients with diabetes were surveyed via convenience sampling on 3 WeChat public accounts. Finally, our sample came from 30 provinces across China. Our research also had several limitations. First, the 1276 sampled patients did not sufficiently represent the large population of patients with diabetes in China. Second, our sampling was not stratified by geographic region, urban or rural location, socioeconomic status, age, or diabetes type. Some selection bias was unavoidable. Although the mean age of our patient sample was comparable with that of the national survey concerning diabetes prevalence in China [47], the proportion of patients with T1DM in our sample was higher than the actual disease proportion of total patients with diabetes [48]. Finally, our sampling was based on the WeChat network. Although WeChat has 1.04 billion monthly active users [49], some people do not use WeChat or surf the internet. Thus, actual app usage might be lower, particularly among elderly patients.

Our study was a cross-sectional survey. Although patients’ perspectives and attitudes are very important when developing a mobile app for their use [50,51], people’s attitudes toward what is useful and what might work are heavily anchored in their present experience regarding the development of technology and how it is implemented. Therefore, these findings must be updated over time as technology develops and people’s perceptions change. Furthermore, many factors influence app use. Although we adjusted for some factors in the multivariate analysis, other potential confounding factors remain.

### Conclusions

Using an exploratory approach, we found that awareness and use of diabetes apps among the Chinese diabetic population and the proportion of diabetologists using diabetes apps to manage patients are low. There are a few differences in the needs for and choice of diabetes apps between patients with T1DM and patients with T2DM. Therefore, designing apps targeted for different patients’ needs and conducting high-quality RCTs will improve the effectiveness of apps, provide evidence for patients to choose suitable apps, and be conducive to the promotion of diabetes apps. China should increase public awareness of diabetes apps, and relevant policies and regulations are needed to support doctors’ use of apps to manage patients. Diabetes app use in China has good potential. Diabetes apps are potentially effective supplements that can be used in traditional outpatient clinics to improve glycemic control in Chinese patients with diabetes.

## Abbreviations

DSMES: diabetes self-management education and support
GDP: gross domestic product
HbA_1c_: glycosylated hemoglobin
HCP: health care professional
IQR: interquartile range
LADA: Latent Autoimmune Diabetes of Adults
mHealth: mobile health
OR: odds ratio
RCT: randomized controlled trial
T1DM: type 1 diabetes mellitus
T2DM: type 2 diabetes mellitus
